# Behaviors of stem cells on carbon nanotube

**DOI:** 10.1186/s40824-014-0024-9

**Published:** 2015-02-02

**Authors:** Ju-Ro Lee, Seungmi Ryu, Soojin Kim, Byung-Soo Kim

**Affiliations:** School of Chemical and Biological Engineering, Seoul National University, Seoul, 151-744 South Korea; Interdisciplinary Program for Bioengineering, Seoul National University, Seoul, 151-744 South Korea; Department of Biomedical Engineering, University of Alabama at Birmingham, Birmingham, AL USA

**Keywords:** Carbon nanotube, Differentiation, Stem cell, Substrate

## Abstract

Regulating stem cell microenvironment is one of the essential elements in stem cell culture. Recently, carbon nanotube (CNT) has come into the spotlight as a biomaterial that retains unique properties. Based on its high chemical stability, elasticity, mechanical strength, and electrical conductivity, CNT shows great potential as an application for biomedical substrate. Also, properties of CNT could be further regulated by appropriate chemical modifications of CNT. Recent studies reported that modulating the cellular microenvironment through the use of CNT and chemically modified CNT as cell culture substrates can affect proliferation and differentiation of various types of stem cells. This review summarizes the unique biological effects of CNT on stem cells.

## Introduction

Stem cells, which have the ability to self-renew and multipotently differentiate into several phenotypes, have been regarded critical for groundbreaking therapy in the field of regenerative medicine. Therefore, strategies to promote their proliferation and control their differentiation are subject of great interest. In tissue engineering, mesenchymal stem cells (MSCs), neural stem cells (NSCs), and embryonic stem cells (ESCs) can be induced to differentiate into various terminally differentiated cells including chondrocytes, osteoblasts, neurons, and myocytes under specific culture conditions. These differentiated cells can be injected directly into damaged tissue or conjugated with specific substrates, and used to regenerate damaged tissues. MSCs, NSCs, and ESCs can be differentiated into mature cells by modulating their cellular microenvironment. One of the most effective ways to control the fate of stem cells is by changing the properties of the cell culture substrates, which can provide dynamic microenvironmental and morphological cues for stem cell proliferation and differentiation. CNT has emerged as a new potential cellular culture substrate that could alter the behavior of stem cells [1].

## Review

This review briefly outlines the unique characteristics of CNT and highlights the recent applications of CNT for tissue engineering through stem cell differentiation. Also, biocompatibility and toxicity of CNT will be discussed in this review.

### Carbon nanotube

**Figure 1:**
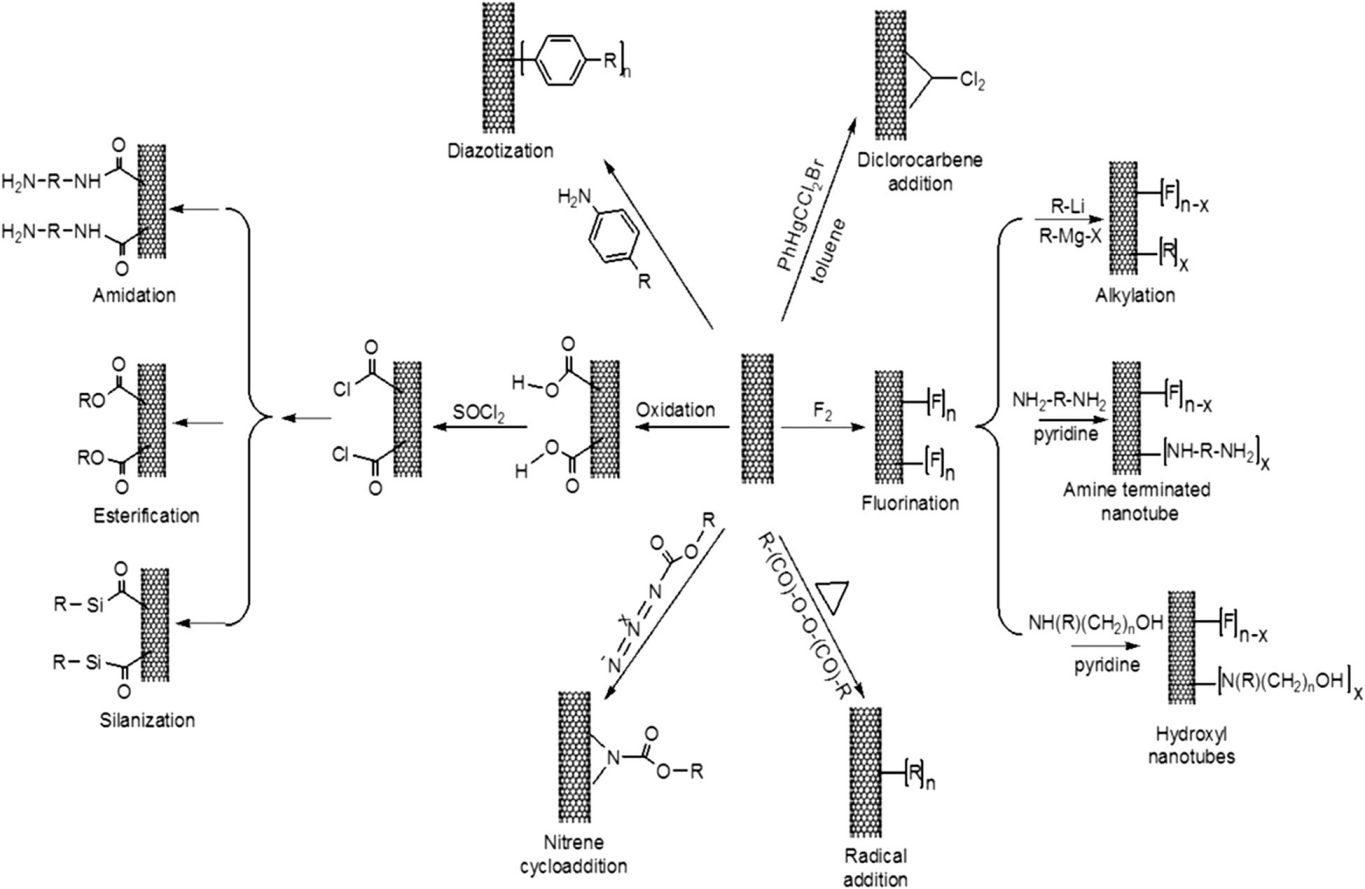
**Surface functionalizaton of CNTs.** Schematic diagram showing the surface chemistry of CNTs. The surface of CNTs can be functionalized with different chemical groups through various chemical reactions. (adapted from reference [11]).

CNT consists of a sheet/sheets of graphitic structure rolled into a cylinder. Due to its hexagonal structure and π electrons conjugation, CNT possesses high mechanical strength, flexibility, and electrical conductivity [2,3]. Mainly three conventional methods have been used for synthesis of CNT such as arc discharge, laser ablation, and chemical vapor deposition methods. Arc discharge and laser ablation methods are methods in which high energy input, such as laser beam, induces the assembly of carbon atoms. However, these have difficulties for large-scale production. In the chemical vapor deposition method, certain catalysts are used to assemble carbon atoms into CNTs. The chemical vapor deposition method can be done under mild condition [4]. CNT can be categorized into single-walled CNT (SWCNT), multi-walled CNT (MWCNT), and functionalized CNT. SWCNT is made up of a single sheet of graphene rolled up, and its ends are closed with fullerene caps. MWCNT is made up of multiple sheets of graphene cylinder. Functionalized CNT is modified CNT with specific organic groups attached on its surface. Its properties can be controlled in many ways as CNT can be easily functionalized [5]. There are two types of CNT surface modifications: non-covalent functionalization and covalent functionalization [6]. Non-covalent functionalization involves coating or dispersion of CNTs on hydrophilic macromolecules such as peptides, single-stranded DNA, and polymers like polyethylene glycol or polyethleneimine (PEI) [7-9]. For example, by mixing CNT with collagen, CNT gains greater resistance for the use of three-dimensional arrays [10]. CNT covalent functionalization is a method that covalently bonds CNT molecules with proteins, surfactants or genetic species. Covalent functionalization changes the physical and chemical properties of CNT, such as its surface charge and reactivity. For example, CNT surface charge can be controlled through addition of functional groups. Figure 1 shows the surface of CNTs with different chemical groups through various chemical reactions [11]. To terminate its ends with negatively charged groups, pristine CNT was refluxed in nitric acid. After removal of the metal catalyst, CNT surface was modified with a carboxylic group, making it a carboxylic functionalized CNT (CNT-COOH). By addition of oxalyl chloride, CNT-COOH can be modified to acyl chloride form, CNT-COCl. For terminating its ends with neutrally or positively charged group, CNT-COCl was functionalized with poly-*m*-aminobenzene sulfonic acid and ethylenediamine, respectively [6]. Currently, by virtue of facile functionalization of CNT, CNT has served as potent therapeutic vectors of genes, drug-delivery vehicle, and culture substrate for stem cell differentiation [12-14].

### Stem cell differentiation on carbon nanotube

Stem cells play an important role in tissue engineering and regenerative medicine because of their ability to self-renew and differentiate. Controlling the fate of stem cells is one of the most studied issues in tissue engineering. As a culture substrate, CNT has drawn tremendous interests in tissue engineering as it has the ability to dynamically direct stem cells lineage. For example, CNT has a high binding affinity to biological molecules such as extracellular matrix (ECM) proteins. Due to its high binding affinity to ECM proteins such as fibronectin, CNT can efficiently control cellular behavior [15]. In addition, as mentioned above, properties of CNT can be easily modified to improve its biocompatibility as a cellular culture substrate. So far, CNT has been a subject of studies for culture of various stem cell lines, such as neural stem cells, embryonic stem cells, and mesenchymal stem cells.

### Neural stem cells

**Figure 2:**
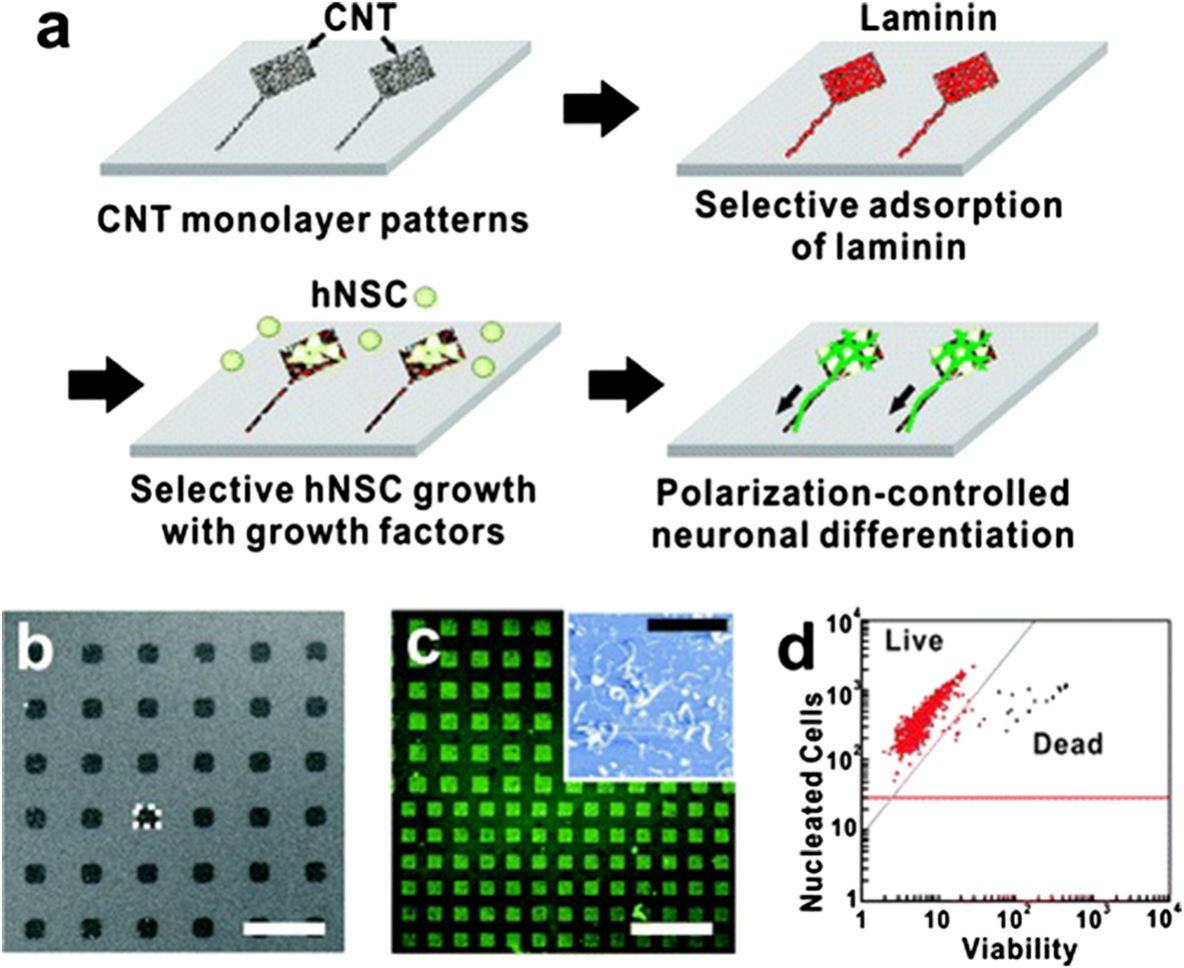
**Shape-controlled CNT substrates for hNSCs growth and polarization. (a)** Schematic diagram showing the process of the polarization-controlled neuronal differentiation of hNSCs. Shape-controlled CNT substrates induced the differentiation of hNSCs into neuronal lineages. **(b)** SEM image of CNTs substrate. Scale bar represents 40 μm. **(c)** Immunofluorescence image of anti-laminin (green) bound to the laminin absorbed on the CNT substrate. Scale bar represents 200 μm. The inset shows AFM image of laminin-coated CNT substrate. Scale bar of the inset represents 2 μm. **(d)** Cell viability of hNSCs cultured on CNT substrates for 3 day proliferation. The data indicates that 98% of hNSCs cultured on CNT substrates were alive (red) (adapted from reference [21]).

The unique mechanical and electrical properties of CNT have been used in biological applications to design conductive substrates, especially in neural tissue engineering [16]. One of the first experiments that applied CNT as a cell culture substrate investigated the efficacy of SWCNT/PEI composite as a NSCs culture substrate [17]. In this study, with the aid of SWCNT/PEI composite, NSCs differentiation toward neurons and oligodendrocytes was enhanced. Not only the neural outgrowth was increased, but also the expression of microtubule-associated protein 2 (MAP-2) was promoted. Moreover, it has been reported before that morphological cues of the cell culture substrate could impose an important effect on behavior of neural cells [18]. Therefore, CNT, which is a nanoscale biocompatible material, has been proposed as NSCs culture substrate for enhancing the neuronal differentiation of NSCs [19,20]. Figure 2 shows that by controlling the shape of the CNT substrates, the growth, polarization, and differentiation of NSCs can be controlled. In shape-controlled CNT substrates, NSCs show better differentiation potential into astroglial and neural cells with increased expression of GFAP and Tuj1 [21].

### Embryonic stem cells

Not only CNT imposes great effects on neuronal differentiation of NSCs, but also it has been reported to promote neural differention of human ESCs (hESCs). When hESCs were seeded onto hydrophilic CNT-poly(acrylic acid) composite, hESCs differentiation toward neuronal lineages was elevated up to two-fold when compared to the hESCs cultured with poly(L-ornithine) (PLO), the conventional standard polymer for culturing neural cells. Moreover, the substrate composite showed no effect on hESCs viability and adhesion [22]. In addition, CNT/collagen composite was reported to promote neural differentiation of hESCs. Type I collagen, which is one of the major component of ECMs that support neuronal cell types, was modified with CNT. Not only CNT improved the biocompatibility of the collagen, but also it heightened the interaction between the collagen compound hESCs cultured on this CNT/collagen composite differentiated into ectodermal lineage in day 3, and into neural lineage in day 6, with enhanced expression of nestin [23]. Nestin is a representative marker that identifies neural stem cells [24]. Also, poly(methacrylic acid)-grafted CNT can greatly enhanced the differentiation of hESCs into neural lineage compared to the hESCs cultured on PLO substrate [25].

### Mesenchymal stem cells

**Figure 3:**
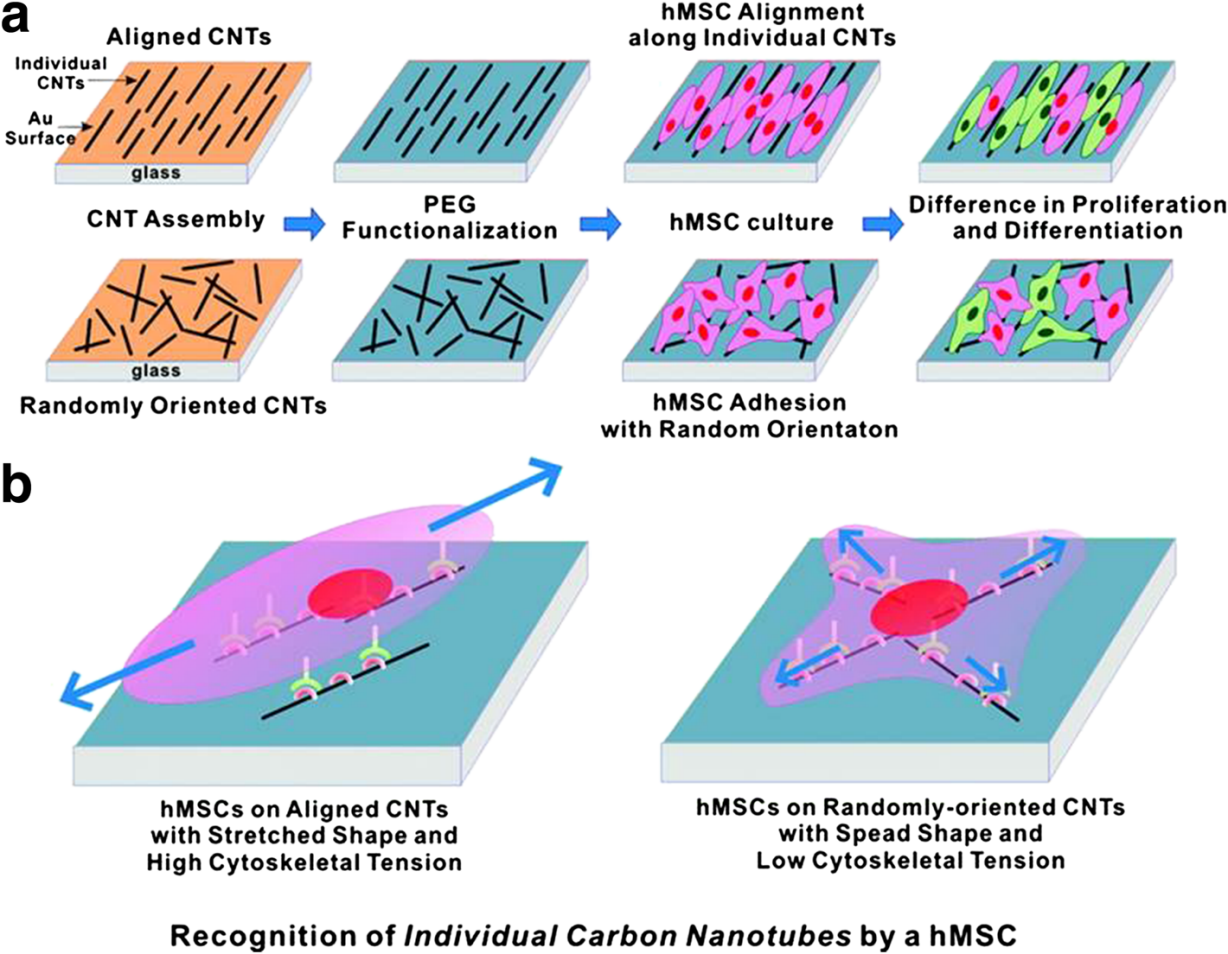
**Growth and differentiation of hMSCs on CNT with the different arrangement of individual CNTs. (a)** Schematic diagram showing the experimental procedure. CNTs were assembled on Au substrates in an aligned or a randomly oriented formation, following functionalization with thiolated polyethylene glycol (PEG-SH). hMSCs were cultured and investigated. **(b)** Plausible model to explain the hMSC responses to the orientation of CNT networks. The hMSCs were elongated along the alignment direction of the CNTs because of a high affinity between CNTs and cells (adapted from reference [28]).

CNT can also promote the differentiation of human mesenchymal stem cells (hMSCs). As a cell culture substrate, CNT was reported to increase the surface roughness of the substrate and promote high adsorption of ECM proteins such as fibronectin and vitronectin [15]. Using such properties of CNT, fibronectin-coated SWCNT substrate was shown to enhance hMSCs spreading compared to the conventional tissue culture plate, promoting neural gene expression such as nestin and MAP-2, a cytoskeletal protein in neurons and dendrites [26,27]. Also, on topological modified CNT substrates, hMSCs show improved cell proliferation and osteogenic differentiation. Square-patterned and aligned CNT substrates promote the expression of core binding factor alpha1, osteocalcin, and alkaline phosphatase which are a transcription factor and an osteoblast specific gene, respectively [28,29]. Figure 3 shows how the topography of CNT substrates surfaces could affect the morphology of hMSCs [28]. In addition, due to electrical properties of CNT, CNT as a cellular substrate can provide electrical stimulation to the cells. One study showed that the electrical current imposed on CNT substrate can enhance the differentiation of hMSCs toward cardiomyocyte lineage [30].

### Biocompatibility and toxicity

Developing a biocompatible and nontoxic substrate that can facilitate stem cells proliferation and differentiation is one of the most pivotal subjects in stem cell research. Pristine CNT shows poor dispersion within most types of solvents. It is insoluble and chemically inert in culture media. CNT alone is rarely used in medical applications, as insoluble CNT among cells can be toxic to the cells [31]. Therefore, surface modification of the CNT is necessary. CNT that has undergone surface modifications could allow higher activity and interaction between the CNT and the cell. In addition, one of the main concerns about CNT composite application on tissue engineering is the harmful immune response against CNT. Coating CNT with biocompatible protein has been the candidate to alleviate the immune response. For example, laminin, an essential part of human ECM, was fabricated with SWCNTs in layer-by-layer structure. SWCNT-laminin films eventually minimized the immune response without affecting neural differentiation potential of CNT. The result implied that CNT-protein composite can be used as a potential biocompatible material for neural tissue engineering [20].

Potential toxicity of CNT has been a considerably important issue for biomedical applications. CNT toxicity depends on its physical and chemical properties, such as CNT dimensional parameter or nature of the attaching target surface. However, there are still no general theories on what makes CNT more or less biocompatible and toxic. Despite numerous studies, it is uncertain to either classify CNT as a toxic or nontoxic material. Although CNT shows toxicity at some degree, it could be mitigated by controlling some of its properties. With sustained research, CNT could be hope for a potential biomedical tool.

## Conclusions

CNT has emerged as a promising biocompatible substrate among researchers for its unique properties. In tissue engineering, it is important for the substrate to mimic the natural environment of stem cells in order to control direction, proliferation, and differentiation of stem cells. In nature, both proliferation and differentiation of stem cells are highly related to external signals and metabolic pathways those are dependent on the ECM. In other words, proliferation and differentiation of stem cells are favorably based on the nanotopography and microenvironment of cell adhesion substrates. CNT could be managed to represent a favorable topography and microenvironment for stem cells. However, there are still various technical shortages that should be investigated for CNT application on cell therapy. The future potential of CNT application is promising for broad types of tissue therapies such as heart, liver, bone, and other tissues.

## Abbreviations

CNT: Carbon nanotube
MSC: Mesenchymal stem cell
NSC: Neural stem cell
ESC: Embryonic stem cell
SWCNT: Single-walled carbon nanotube
MWCNT: Multi-walled carbon nanotube
PEI: Polyethyleneimine
CNT-COOH: Carboxylic functionalized carbon nanotube
ECM: Extracellular matrix
MAP-2: Microtubule-associated protein 2
hESC: Human embryonic stem cell
PLO: Poly(L-ornithine)
hMSC: Human mesenchymal stem cell
GFAP: Glial fibrillary acid protein
